# “I Am Afraid of Positioning my Baby in Prone”: Beliefs and Knowledge about Tummy Time Practice

**DOI:** 10.1155/2023/4153523

**Published:** 2023-04-19

**Authors:** Bianca Fernandes Vasconcelos e Silva, Sabrinne Suelen Santos Sampaio, Julia Raffin Moura, Cléa Emanuela Barreto de Medeiros, Carolina Daniel de Lima-Alvarez, Camila Rocha Simão, Ingrid Guerra Azevedo, Silvana Alves Pereira

**Affiliations:** ^1^Post-Graduate Program in Rehabilitation Sciences, Federal University of Rio Grande do Norte, Brazil; ^2^Anita Garibaldi Center for Education and Research in Health, Santos Dumont Institute, Brazil; ^3^Physiotherapy Department, Federal University of Rio Grande do Norte, Brazil; ^4^Post-Graduate Program in Sciences of Rehabilitation, University of Brasilia, Brazil; ^5^Research Directorate, Catholic University of Temuco, La Araucanía, Chile

## Abstract

**Objective:**

To identify beliefs and knowledge about tummy time (TT) practice and its repercussions on motor development.

**Methods:**

Longitudinal study carried out with parents/caregivers of infants older than 30 days of life. Two assessments were performed. A structured interview was conducted, while the babies were between one and six months old to identify beliefs, knowledge about TT, and the motor milestone achievement expected for the age. At six to 12 months, the risk of motor development delay was tracked using the survey of well-being of young infant questionnaire (SWYC).

**Results:**

41 families responded to the SWYC questionnaire (21 were allocated to the TT group). 31.70% reported that it was not important to put the infants in a prone position while awake, and 70.70% said they are afraid their babies would become breathless when positioned in a prone position. 85.70% of infants from the TT group showed typical development, while 55% of the control group showed atypical development for their age (*p* = 0.01). Only three infants from the control group were at risk of delayed motor development (*p* = 0.10).

**Conclusions:**

Most of the families feel insecure about proning their babies and fear breathlessness when positioned. Acquisition of motor milestones prevailed in the TT group, suggesting an association between TT practice and motor milestone achievement.

## 1. Introduction

Motor milestone achievement in the first years of life reflects the maturation and development of the central nervous system and is affected by intrinsic [1] and extrinsic factors (i.e., infant characteristics, environment, and stimuli) [2]. In the first months of the infant's life, early identification of developmental delay enables effective interventions due to their greater brain plasticity [3].

A way to prevent developmental delay in newborns is providing infants with adequate stimuli with tummy time (TT), encouraged and supervised by an adult [4]. TT or awake-prone positioning is a form of physical activity recommended for infants <6 months of age [5]. Prone positioning relieves pressure in the posterior region of the head, stimulates core muscles, and improves the force of trunk and cervical extensor muscles and thoracic/scapular mobility [6]. In addition to promoting muscular development, TT is also positively associated with mental and social skills, as well as allowing the infant to visually explore their environment and reduced BMI [7–10].

In this sense, TT practice is included in most guidelines on infant health since it correlates with positive long-term outcomes (i.e., reduced television use, increased “active playing” time, and reduced childhood obesity) [8–10]. Its daily practice is widely recommended for no prone sleeping and supervised awake prone time, starting at birth (two to three times daily, from three to five minutes) and gradually evolving up to the age of six months for at least 30 minutes daily [11–14].

Because studies have demonstrated a positive association between TT and the infant's development, [10, 15–17] it has been a component of guidelines in different countries such as Australia, the United Kingdom, and Canada [13, 18, 19] as well as a component of National Academy of Medicine Guidelines [20] and the American Academy of Pediatrics [11]. However, evidence shows that some parents/caregivers are not aware of this practice [21]. Therefore, the aim of this study is (1) to identify beliefs and knowledge about TT practice and (2) its repercussions on motor development.

## 2. Material and Methods

This longitudinal study was conducted at an education and research center located in Northeastern Brazil. All parents/caregivers received verbal and written explanations regarding study objectives and methodology and signed an informed consent form. This study was approved by the research ethics committee (resolution 466/12 from the National Committee of Ethics in Research) and performed according to the Declaration of Helsinki.

A convenience sample of parents/caregivers was recruited between October and December 2019, in a childcare ambulatory. Parents/caregivers of full-term infants aged > 30 days were included. Those who did not complete the survey of well-being of young children (SWYC) questionnaire were excluded. Although there are several instruments available for identifying signs of risk for developmental delays, the SWYC is particularly advantageous due to its ease of use as a first-line screening tool and its ability to assess children following age-specific domains [22].

Sample size was calculated (G^∗^ Power software, version 3.1.9.4), considering the risk of cranial asymmetry in infants with (25.8 ± 21.4) and without (48.7 ± 28.9) reduced time in prone (6). With Cohen's d effect size of .90, a power of .80, and *α* error of .05, the minimal number of infants was estimated as 38 infants (total sample).

A structured interview was conducted with all parents/caregivers while their babies were between one and six months old to collect familiarity of parents with prone positioning (TT practice), sociodemographic data regarding parents/caregivers (education and occupation of parents), pregnancy, and infant data (complication during pregnancy, type of delivery, gender, gestational age, birth weight, Apgar, hospitalization, and use of mechanical ventilation), and investigation about other infant products/services as smart steps/walkers or sessions of multiprofessional team (occupational therapist, physiotherapy, psychology, or speech therapy).

Motor development was assessed between one and six months by motor milestone achievement expected for the age of six to 12 months old using the SWYC.

TT practice was investigated using a structured questionnaire composed of four domains. Domain #1 is about where and who cared for the infant. Domain #2 assessed the approach of parents and infants related to TT practice. In this domain, we investigated frequency and time dedicated to prone positioning (zero to 30 minutes or more), starting age, place where it is usually performed (e.g., bed, crib, pillow, floor, or mother's lap), stimuli (e.g., toy, voice, television, or mirror), infant reaction (e.g., cry, look uncomfortable, calm, or smile), perception (ease, security, or importance), and parental feeling and confidence regarding TT practice and factors influencing decision-making. For the latter, we presented concepts to parents/caregivers and asked if they agreed or not with the statements. Infants whose parents reported setting aside time to perform prone positioning daily were allocated to the TT group, and the remaining infants were allocated to the control group. Domain #3 evaluated the knowledge of parents/caregivers regarding TT using statements classified as “true” or “false,” while Domain #4 identified where the knowledge was acquired. The interview lasted about 10 minutes and was conducted in a private environment.

To track the motor milestone achievement expected for the age, a set of figures representing possible motor skills was presented to parents/caregivers and asked if the infant could perform them. It was considered a “typical infant” when motor milestones expected for his/her age were achieved, or “atypical infant” when he/she did not achieve the motor milestones expected for the age. The key milestone expected for infants aged between one and three months was to lift the head in the prone position, whereas rolling alone was considered for infants aged between four and six months [23].

The risk of motor development delay was tracked using the SWYC questionnaire between the age of six to 12 months. The moments of the evaluations were carried out according to the three stages of motor development determined by each corresponding SWYC form (6-8 months, 8-11 months, and 12 months). For this, a telephone interview was conducted with parents/caregivers responsible for the infant's care. SWYC is a free, fast, and easy online tool to monitor infants' development aged between one and 65 months. It was recently validated for the Brazilian population and presents three major domains: motor development, family's behavior, and risk factors (i.e., depression, conflicts between parents, food insecurity, and illicit drug abuse) [24, 25].

For this study, only the “developmental milestones” domain was applied, which investigates the infant's behavior using 10 questions according to age group. [24] The study had 25 forms for 6 months to 8 months, 15 forms for 9 to 11 months, and 1 form to 12 months. Responses were classified as “not yet,” “somewhat,” or “a lot” for each question. “Risk” or “without risk” classifications followed the scoring system proposed by Moreira et al. (2018) [24], which considers [25].

GraphPad Prism software version 6 (La Jolla, USA) was used for statistical analyses. Data normality was verified using the Kolmogorov-Smirnov test. The Mann–Whitney test was used to compare data between groups, while the Chi-square test investigated associations between TT and both, the motor milestones and risk of developmental delay. Data are shown as median, 25-75% interquartile range, and 95% confidence interval (95% CI). A *p* value of <0.05 was considered significant for all statistical analyses.

## 3. Results

Fifty-nine parents/caregivers were interviewed about TT and 41 responded to the SWYC questionnaire at the age of six to 12 months. Of the 41 infants included, 21 were allocated in the TT group, five (12.20%) had been hospitalized, three (7.30%) were on mechanical ventilation during the neonatal period, 34 (82.90%) attended monthly medical consultations, and six (14.60%) were followed up weekly by a professional from the multiprofessional team (occupational therapist, physiotherapy, psychology, or speech therapy). No infants used smart steps or walkers.  Table 1 shows sample characteristics.

Most parents are afraid of positioning the baby in prone (70.70%) and 31.70% reported that positioning infants in prone while awake is not that important. Factors related to decision-making on TT practice are shown in Table 2.

In the TT group, twelve infants (57.10%) remained in prone positioning for one to five minutes daily, eight (38.10%) infants remained in six to eleven minutes in the position, and only one (4.80%) remained in prone for more than thirty minutes. Almost 52.40% of the studied families feel confident positioning the baby in prone. In the control group, eleven infants did not achieve motor milestones at six months of age, and three of them were at risk of motor development delay as assessed by SWYC at 12 months of age. Associations between TT and motor milestones are presented in Table 3.

## 4. Discussion

Data suggest that infants aged between one and six months practicing prone positioning daily are more likely to achieve motor skills expected for their age, such as antigravity control of cervical muscles and rolling. However, infants who did not practice prone positioning daily were not at risk of developmental delay at the age of six to 12 months.

Corroborating partially with these results, in Taiwan, Kuo et al. (2008) followed 288 infants and found that rolling and crawling acquisitions at the age of four to 24 months occurred significantly early in the group that practiced TT [26]. Kuo et al. and Dudek-Shriber and Zelazny (2007), in a study conducted with 100 infants at the age of four months, observed an association between time in prone and skill gains in supine, prone, and seated positions, suggesting that TT enables motor skill development and weight unloading patterns in all positions [26, 27] Furthermore, head lifting is also increased in children practicing TT [28], while parental participation and interaction with toys may enhance child tolerance to the method, allowing more time in prone and greater cervical muscle activation [15].

Although different studies corroborate that daily practice of TT can contribute to motor milestone achievement [9, 26, 27] and reduce the risk of delayed motor development, [29] the time dedicated to the method does not seem to be a consensus [9, 26, 27]. In our study, of 21 families in the TT group, eight spent six to eleven minutes practicing prone positioning daily, twelve practiced between one to five minutes, and only one remained in the posture for more than 30 minutes. Dudek-Shriber and Zelazny (2007) suggest at least one hour and 21 minutes of daily practice, while Koren et al. (2019) recommended more than seven minutes [9, 27] The American Academy of Pediatrics [30], the Australian Guidelines for Physical Activity [12], the United Kingdom Physical Activity Guidelines [13], and World Health Organization [14] suggest starting with a minimum of six minutes and gradually evolve to 30 minutes daily.

Kuo et al. (2008) demonstrated that 20 minutes was reasonable for TT practice and suggested time does not influence the motor milestone achievement [26]. Many parents do not understand the risks that are associated with limited tummy time during the early months of life [6, 21].

We believe that TT offers the infant a range of possibilities regardless of daily practice since it increases the visual field [31]. Besides, it provides several new stimuli, allowing greater articular amplitudes, gains in stability (cervical, thoracic, and scapular girdle) [23] and muscle strength [28], and weight unloading and transference (i.e., extrinsic factors influencing motor milestone achievement). The time observed in our study was also sufficient to allow associations between prone positioning and milestone achievement expected for the age.

Other studies reinforce that adaptation of information to local language and culture contributes to adherence, while lack of access to information, especially in poorer and underprivileged families, contributes to delay the dissemination of such practice [32–34]. Ricard and Metz (2014) highlight that insecurity probably contributes to low adherence [35].

In order to determine strategies to support parent's implementation of TT, occupational therapists have provided a new model to plan educational programs [35]. The planning and implementation of the interventions are guided by the application of an established framework informed by parental perspectives. The primary barriers to the implementation of tummy time reported by parents included the baby not tolerating TT; remembering to implement TT; being busy and finding the time; and limited confidence in knowledge [35]. Our results corroborate with this finding since almost half of the families in the TT group did not feel confident in proning their babies.

From the perspective of motor development, only three infants from the control group presented SWYC scores indicating risk. Despite this, the absence of prone positioning practice was not associated with the risk of developmental delay, probably because families from the control group may have experienced prone positioning during the follow-up interval. Thus, the lack of strict follow-up may have impaired data interpretation, and it characterizes a limitation of this study. Previous studies performed in developed countries showed that low adherence is reversed after adopting and disseminating policies encouraging TT practice [5, 33, 35]. Adherence rates in the United States rose from 34% to 100% after incentive campaigns [33]. In Brazil, insufficient data and campaigns related to the practice of TT revealed this gap. However, participation in the first interview may have aroused families' curiosity regarding prone positioning; thus, it could have influenced the results of the second assessment. We recognize that difficulty in monitoring prone positioning practice between assessments (six-month interval) may have hindered associations between the method and risk of developmental delay at the age of six to 12 months, which may be considered a limitation of this study. Thus, further studies are needed to elucidate this point.

## 5. Conclusions

Data suggests that infants aged between one and six months practicing prone positioning daily are more likely to achieve motor skills expected for the age, such as antigravity control of cervical muscles and rolling. Considering the demographic, socioeconomic, and behavioral particularities of the Brazilian context, the benefits pointed out in this study may motivate new, larger, and further studies, contributing to public policies and disseminating and implementing the TT practice.

## Figures and Tables

**Table 1:** Characterization of infants (*n* = 41).

	Total	Tummy time (*n* = 21)	Control (*n* = 20)		*p*
Data of parents and/or caregivers					
Education Incomplete high school Complete high school	22 19	9 12	13 7	*X* ^2^ 2.02	0.21
Occupation					0.71
Home occupation	32	17	15	*X* ^2^ 0.21	
Other jobs	9	4	5		
Siblings					0.53
Yes	23	13	10	*X* ^2^ 0.58	
No	18	8	10		
Pregnancy data					
Complication during pregnancy					1.00
Yes	30	15	14	*X* ^2^ 0.06	
No	11	6	4		
Type of delivery					0.34
Vaginal	26	13	11	*X* ^2^ 1.19	
Cesarean	15	8	9		
Infant data					
Gender					0.75
Female	20	11	9	*X* ^2^ 0.22	
Male	21	10	11		
GA (weeks)	39 (38–40)	39 (38–40)	39 (38–41)	U 174.0	0.32
Apgar (5^th^ minute)	9 (9–9)	9 (9–10)	9 (9–9)	U 139.5	0.08
BW (grams)^∗^	3,397.78 (±484.20)	3,219.71 (±417.19)	3,594.58 (±486.62)	*t* 2.62	0.01^∗^
Not lift the head in prone position between 1 to 3 months	5	2	3	*X* ^2^ 0.22	0.67
Not roll alone between 4 to 6 months	3	0	3		

Note: data are shown as absolute frequency, median, and 25-75% interquartile range. GA: gestational age at birth; BW: birth weight; DL: days of life. *X*^2^: Chi-square test; U: Mann–Whitney *U*-test; *t*: Student's *t*-test. ^∗^*P* ≤ 0.05.

**Table 2:** Factors related to decision-making on tummy time practice in the first interview.

Affirmations	Total (*n* = 41)	Tummy time (*n* = 21)	Control (*n* = 20)		*p*
“It is dangerous to position infants prone”	23 (56.10%)	8	15	*X* ^2^ 5.66	0.02^∗^
“I am afraid my baby become breathless when positioned prone”	29 (70.70%)	14	15	*X* ^2^ 0.34	0.73
“I am afraid my baby will fall asleep in prone position”	15 (36.60%)	8	7	*X* ^2^ 0.04	1.00
“I feel ‘sad' when my baby cries during prone positioning”	21 (51.20%)	10	11	*X* ^2^ 0.22	0.75
“I am too busy (I do not have time) to position my baby prone while awake”	6 (14.60%)	2	4	*X* ^2^ 0.90	0.41
“Positioning infants prone while awake is not that important; most infants are fine”	13 (31.70%)	5	8	*X* ^2^ 1.24	0.32
“I feel confident positioning my baby prone”	14 (34.10%)	11	3	*X* ^2^ 6.36	0.02^∗^
“I know a lot about how, when, and for how long to position my baby prone”	19 (46.30%)	11	8	*X* ^2^ 0.63	0.53
“I know where to get answers about positioning my baby prone”	21 (51.20%)	12	9	*X* ^2^ 0.60	0.53
“It is only safe for the baby to be positioned prone when he/she can roll on their own”	17 (41.50%)	7	10	*X* ^2^ 1.17	0.35
“The baby can be positioned prone even though he/she cannot lift his head”	17 (41.50%)	7	10	*X* ^2^ 1.17	0.35
“When infants are positioned prone for more than five minutes, it can cause some harm”	10 (24.40%)	5	5	*X* ^2^ 1.17	1.00

Note: absolute and relative (%) number of parents/caregivers who responded “I agree” to questions presented in D3; *X*^2^: Chi-square test; *p* value for Chi-square test. ^∗^*p* ≤ 0.05.

**Table 3:** Association between tummy time and risk of motor development delay using the survey of well-being of young children (SWYC).

	Tummy time group	Control group		*p*
Typical development	18 (85.70%)	9 (45.00%)	*X* ^2^ 7.55	<0.001^∗^
Atypical development	3 (14.30%)	11 (55.00%)		

Note: *X*^2^: Chi-square test; *p* value for the Chi-square test. ^∗^*p* ≤ 0.05.

## Data Availability

The data is available on request from the corresponding author.

## Additional Points

*Highlights*. What is known about the subject? (i) Prone positioning relieves pressure in the posterior region of the head, stimulates core muscles and improves force of trunk and cervical extensor muscles. (ii) Tummy Time practice is included in most guidelines on infant health since it correlates with positive long-term outcomes. What this study adds? (i) Most parents are afraid of positioning the baby in prone. (ii) Parents' confidence can influence adherence to the prone positioning practice. (iii) Families feel insecure about proning their babies.
